# Comparative effectiveness of hand hygiene modalities in reducing microbial colonization on nurses’ hands in surgical settings in Jordan

**DOI:** 10.3389/fpubh.2026.1794667

**Published:** 2026-05-08

**Authors:** Hanadi Dakhilallah, Bander Saad Albagawi, Kamlah Ahmed Sari Al-Olaimat, Rasha Abdulhalim Alqadi, Mohammad Ahmad Al-Me’ani, Mohamed MI Helal, Boshra Karem Mohamed El-Sayed

**Affiliations:** 1 Department of Nursing Administration and Education, College of Nursing, Imam Mohammad Ibn Saud Islamic University (IMSIU), Riyadh, Saudi Arabia; 2 Department of Medical Surgical, College of Nursing, Imam Mohammad Ibn Saud Islamic University, Riyadh, Saudi Arabia; 3 Department of Maternal and Child Health Nursing, Al al-Bayt University, Mafraq, Jordan; 4 Department of Medical-Surgical Nursing, North Private College of Nursing, Arar, Saudi Arabia; 5 Department of Clinical Nursing, Faculty of Nursing, Middle East University, Amman, Jordan; 6 Department of Basic Sciences and Preparatory Year, North Private College of Nursing, Arar, Saudi Arabia; 7 Department of Nursing Administration, Faculty of Nursing, Alexandria University, Alexandria, Egypt

**Keywords:** alcohol-based hand rub, and infection prevention, hand hygiene, hand washing, microbial colonization, surgical nurses

## Abstract

**Objective:**

To evaluate and compare the effectiveness of hand washing, alcohol-based hand rub, and their combination in reducing microbial colonization on nurses’ hands in surgical departments.

**Methods:**

A quasi-experimental comparative study was conducted at Jordan Hospital, a tertiary private healthcare institution in Amman, Jordan across 10 surgical departments. Forty-five nurses were alternating sequence as participants were assigned into three equal groups: G1 (hand washing only), G2 (alcohol-based hand rub only), and G3 (hand washing followed by hand rub). Four tools were used: (1) socio-demographic and medical data, (2) infection prevention and control knowledge assessment, (3) hand hygiene observational checklist, and (4) microbiology laboratory assessment. Nurses’ knowledge, hand hygiene practices, and microbial colonization were evaluated at baseline (pre-intervention), post–1st week, post–2nd week, and post–3rd week.

**Results:**

Pre-intervention, the majority of nurses demonstrated insufficient knowledge regarding infection prevention and hand hygiene. Post-intervention assessments showed significant improvements in knowledge across all groups. Hand hygiene compliance significantly increased in G2 and G3 at both post-intervention time points, while G1 exhibited improvement only after the first week. Microbial colonization on nurses’ hands decreased significantly in all groups, with G3 showing the greatest reduction. The combined modality (hand washing followed by alcohol-based hand rub) was more effective in reducing microbial load than either method alone.

**Conclusion:**

Hand hygiene significantly reduces microbial colonization on nurses’ hands, with the combination of hand washing and alcohol-based hand rub showing greater effectiveness in this study. However, given the limited sample size and short study duration, these findings should be interpreted cautiously. Further research with larger samples is needed before making definitive recommendations for routine practice.

## Introduction

Healthcare workers (HCWs) are routinely exposed to a wide range of infectious agents during clinical care, including blood-borne viruses, bacteria, fungi, and other microorganisms. These exposures occur during surgical procedures, routine nursing care, laboratory specimen handling, waste management, and emergency interventions (1, 2). Such occupational risks not only threaten HCWs’ health but also facilitate the transmission of healthcare-associated infections (HCAIs) to patients and other healthcare personnel. Consequently, effective infection prevention and control (IPC) practices—particularly hand hygiene (HH)—are essential to interrupt cross-transmission.

HCAIs remain a major global public health concern with substantial clinical and economic consequences. In the United States, approximately 1.7 million hospitalized patients acquire HCAIs annually, resulting in more than 98,000 deaths (3). Similarly, in Europe, more than 4 million patients are affected each year, with nearly 37,000 attributable deaths (4). Beyond increased morbidity and mortality, HCAIs contribute to prolonged hospital stays, increased healthcare costs, and the spread of antimicrobial resistance (AMR) (5, 6). These challenges highlight the critical need for effective and evidence-based IPC strategies.

Hand hygiene is widely recognized as the most effective and cost-efficient measure for preventing HCAIs and limiting the transmission of antimicrobial-resistant organisms. Its importance was further reinforced during the COVID-19 pandemic, which highlighted HH as a key preventive measure against emerging infectious diseases (7, 8). International guidelines consistently emphasize the importance of optimal HH practices in high-risk clinical settings such as surgical wards, intensive care units, and emergency departments.

Different HH modalities are used in clinical practice, including hand washing with soap and water and alcohol-based hand rubs (ABHRs). Each method has a distinct antimicrobial spectrum depending on its formulation and application technique (9–13). Hand washing primarily removes microorganisms through mechanical action, whereas ABHRs provide rapid and broad-spectrum antimicrobial activity and are widely recommended for routine care due to their convenience and efficiency. However, certain pathogens—particularly spore-forming organisms such as *Clostridium* and *Bacillus* species—may exhibit resistance to alcohol-based preparations, making hand washing essential in situations involving spore contamination (10, 11). The growing burden of antimicrobial-resistant infections, estimated at 2.8 million cases annually in the United States alone (9), further underscores the importance of optimizing preventive strategies such as HH.

Despite strong evidence supporting HH effectiveness, compliance among HCWs remains suboptimal worldwide and is associated with increased HCAIs and compromised patient safety (14, 15). Although education and training programs may improve HH knowledge and short-term adherence, sustaining long-term behavioral change remains challenging (16, 17).

Nurses play a central role in IPC due to their continuous patient contact and frequent involvement in invasive procedures. Their adherence to HH guidelines is therefore critical to reducing infection transmission in healthcare settings (18, 19). However, limited comparative evidence exists regarding the relative effectiveness of different HH modalities in reducing microbial contamination, particularly in high-risk environments such as surgical departments.

Although WHO guidelines (2009) recommend ABHR as the preferred method for routine hand hygiene when hands are not visibly soiled, and hand washing with soap and water when hands are visibly dirty or contaminated, limited evidence exists on whether a sequential combination of hand washing followed by ABHR offers additional microbial reduction. Mechanistically, hand washing removes visible debris, organic matter, and microorganisms that may reduce the efficacy of ABHR, while ABHR provides rapid, broad-spectrum antimicrobial activity (10, 11, 12, 13). In high-risk clinical settings such as surgical departments—where nurses frequently encounter diverse microbial flora and potential contaminants—the combined approach may theoretically enhance microbial clearance compared with either method alone. This study therefore aimed to empirically evaluate whether the sequential application of hand washing and ABHR reduces microbial colonization on nurses’ hands more effectively than either modality alone, while acknowledging that findings are intended to complement, not override, existing WHO recommendations.

Therefore, this study aimed to evaluate the effectiveness of hand washing, alcohol-based hand rub, and their combined application in reducing microbial colonization on nurses’ hands. The findings may contribute to strengthening evidence-based HH practices and supporting infection prevention strategies in surgical settings.

## Aim of the study

To evaluate and compare the effectiveness of hand washing, alcohol-based hand rub, and their combination in reducing microbial colonization on nurses’ hands in surgical departments.

## Research hypotheses

*H1*: Nurses using combined hand washing and alcohol-based hand rub (G3) will demonstrate significantly lower microbial CFU counts than those using hand washing alone (G1).

*H2*: Nurses using combined hand washing and alcohol-based hand rub (G3) will demonstrate significantly lower microbial CFU counts than those using alcohol-based hand rub alone (G2).

*H3*: Nurses using alcohol-based hand rub alone (G2) will demonstrate significantly lower microbial CFU counts than those using hand washing alone (G1).

## Materials and methods

### Research design

A quasi-experimental comparative study was utilized to fulfill the study aim.

### Settings

This study was conducted at Jordan Hospital, a tertiary private healthcare institution in Amman, Jordan in 10 surgical departments, which includes; Hepato-biliary and Pancreatic, Cardiothoracic, Vascular, Oncology, Gastrointestinal, Urology, Ophthalmology, Neurosurgery, Colorectal and ENT. Each department contains two patients’ rooms, one for male and the other for female with a total bed capacity of 200 beds as shown in Table 1.

**Table 1:** Surgical departments affiliated nurses and patient bed number.

Surgical departments	Nurses NO	Bed NO	
		Female	male
1-Hepato-biliary and pancreatic	5	12	12
2-Cardiothoracic	6	10	10
3-Vascular	5	10	10
4-Oncology	6	10	10
5-Gastrointestinal	5	10	10
6-Urology	5	9	11
7-ENT	5	9	9
8-Eye	5	10	10
9-Neuro	5	10	10
10-Colorectal	5	9	9
Total	52	99	101

### Participants and sampling

Nurses who met the inclusion criteria were sequentially assigned to one of three intervention groups: hand washing alone (G1), alcohol-based hand rub (ABHR) alone (G2), or the combination of hand washing followed by ABHR (G3). Assignment was performed using alternating sequence as participants were enrolled, ensuring approximately equal group sizes. No allocation concealment was implemented, and participants and study personnel were aware of group assignments.

### Inclusion and exclusion criteria

Eligible participants were registered nurses working in surgical departments who had at least 6 months of clinical experience and were directly involved in patient care. Participants were required to be available during the study period and willing to provide informed consent.

Exclusion criteria included nurses with active skin conditions affecting the hands (e.g., dermatitis, wounds, or infections), those currently using systemic or topical antimicrobial agents that could influence hand microbial flora, and those who were absent during any phase of data collection or intervention.”

### Sample size

**Figure 1:**
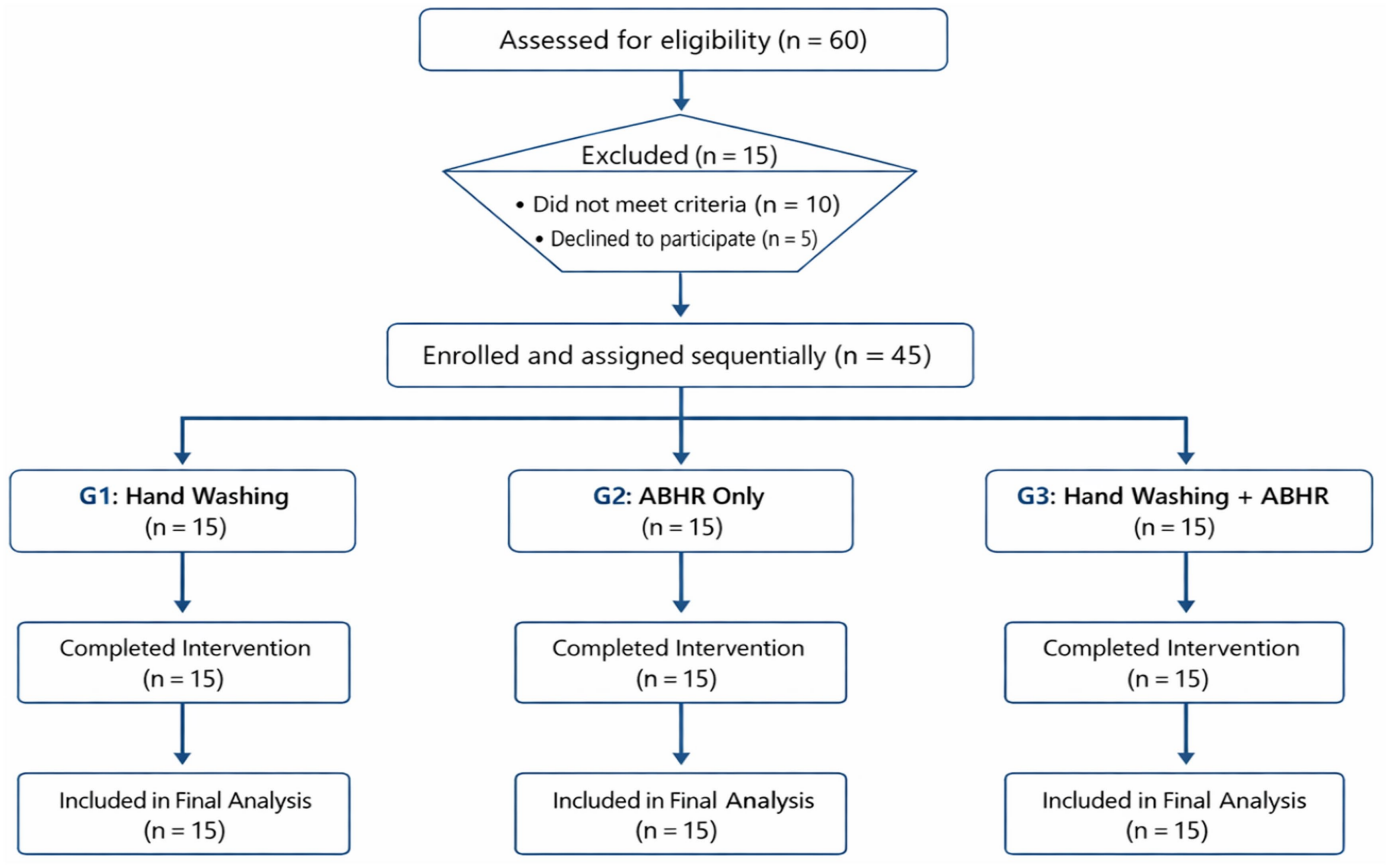
A participant flow diagram.

*A priori* sample size calculation was performed using G*Power 3.1.9.7 for a three-group comparison of repeated measures, assuming a small effect size (*f* = 0.25), *α* = 0.05, and power = 0.80. The calculation indicated that a minimum of 60 participants (20 per group) would be required to detect significant differences in microbial colony counts, which is slightly below the recommended sample size. We acknowledge that this sample may be underpowered to detect small between-group differences, particularly given repeated measurements and microbiological variability. This limitation is addressed in the Limitations section and reflected in the participant flow diagram (Figure 1).

A participant flow diagram (Figure 1) illustrates the number of nurses assessed for eligibility, enrolled, assigned to each group, completed the intervention, and included in the final analysis. The absence of true randomization and allocation concealment is acknowledged as a potential source of selection bias, which is addressed in the Limitations section.

### Study instruments

Four tools were utilized for data collection, developed based on an extensive literature review to assess nurses’ socio-demographic characteristics, knowledge, hand hygiene practices, and microbial contamination.

### Tool I: nurses’ socio-demographic and medical data

Developed from (20). This tool collected baseline information on nurses’ age, gender, education, experience, work unit, and participation in infection control training. Medical data included vital signs, skin conditions of the hands, current or past infections, and antibiotic therapy.

### Tool II: infection prevention and control knowledge assessment

Adapted from (21, 22), this 30-item multiple-choice questionnaire assessed nurses’ IPC and hand hygiene knowledge. Part 1 (15 items) focused on infection control principles, chain of infection, standard precautions, and disease-specific protocols. Part 2 (15 items) assessed hand hygiene concepts, indications, procedures, and types of disinfectants. Correct responses were scored (1), incorrect or missing responses scored (0). Scoring system: Knowledge scores were calculated as the percentage of correct responses out of the total 30 items. A score of ≥90% was classified as satisfactory knowledge, reflecting the high level of infection prevention and control competence expected among healthcare professionals working in surgical settings. This threshold was selected to emphasize optimal knowledge levels required for safe clinical practice.

### Tool III: hand hygiene observational checklist

Based on Murray et al. (23) and Government of South Australia (24), this checklist evaluated nurses’ hand hygiene practices. Part 1 assessed 12 steps of proper hand washing with soap and water; Part 2 evaluated 9 steps of effective alcohol-based hand rub use. Each correctly performed step scored 2, otherwise 0; total scores ≥90% indicated satisfactory practice.

### Tool IV: microbiology laboratory assessment

Developed from Black and Black (25) and Murray et al. (26), this tool measured microbial colonization on nurses’ hands. Data included hand hygiene method, timing, incubation of blood agar plates, colony-forming units (CFU), and organism identification. Microbial growth was categorized as heavy (≥100 CFU), moderate (50–<100 CFU), mild (<50 CFU), or none (0 CFU).

These instruments together provided a comprehensive evaluation of nurses’ demographics, knowledge, compliance with hand hygiene, and the impact on microbial contamination.

### Validation and reliability of study tools

Following the translation of the instruments, content validity was evaluated by a panel of five academic experts from the College of Nursing at Alexandria University. The items were reviewed for relevance, clarity, and cultural appropriateness and modifications were made based on the experts’ feedback and recommendations. Subsequently, a pilot study was conducted with 10% of the target sample to assess the clarity of the items and determine the average time required to complete the instruments. The pilot results confirmed that the items were clearly understood. Participants in the pilot study were excluded from the final sample.

### Reliability analysis

Reliability analysis was performed to assess the internal consistency of the study instruments. Reliability of all tools were tested using Cronbach alpha test. Reliability coefficients for tool I, II, IV were 0.95, 0.90, 0.93, and 0.96, respectively. These results confirm that the scales and their respective dimensions reliably measured the intended constructs.

The hand hygiene practice checklist (Tool III) consisted of structured items assessing the correct performance of procedural steps during hand hygiene. Observations were conducted by a single trained researcher using a standardized observation protocol to ensure consistency in data collection. Because only one observer conducted the assessments, inter-rater reliability was not applicable in this study.

### Data collection procedure

Data collection was conducted between February and October 2025 and included several phases: participant recruitment, baseline assessment, delivery of the educational sessions, implementation of the hand hygiene interventions, and follow-up measurements. Although the intervention for each participant lasted approximately 2 weeks with an additional 3-week follow-up, recruitment and data collection were conducted in a staggered manner across the 10 surgical departments to accommodate nurses’ work schedules and minimize disruption to clinical services. Microbiological processing, data verification, and completion of follow-up assessments also contributed to the overall study timeline.

### Assessment phase

Baseline data were obtained from nurses in the three study groups using four tools: socio-demographic and medical data questionnaire, infection prevention and control and hand hygiene each nurse was interviewed individually, and hand hygiene practices were observed prior to intervention. For microbiological evaluation, nurses imprinted their dominant hand on prepared blood agar plates for 5 s immediately before and after performing the assigned hand hygiene method. Plates were incubated aerobically at 37 °C for 48 h, and microbial growth was quantified as colony-forming units (CFU) by a microbiologist.

### Planning phase

An educational intervention was developed based on baseline findings and current literature. A structured instructional booklet addressing infection prevention and hand hygiene techniques was prepared and distributed according to group assignment. Learning objectives and expected outcomes were defined, and teaching content was sequentially organized.

### Educational standardization

Prior to implementing the hand hygiene interventions, all participants attended three standardized educational sessions on infection prevention and hand hygiene practices. The purpose of these sessions was to ensure a consistent baseline level of knowledge and technique across all participants before evaluating the microbiological effectiveness of the different hand hygiene modalities. The educational content was identical for all groups and was delivered before group allocation to minimize variability in hand hygiene technique (35).

### Implementation/intervention phase

The intervention was delivered in three sessions (45–60 min each) over three consecutive days. Nurses were randomly assigned to one of three groups: hand washing, alcohol-based hand rub, or combined hand hygiene. Two sessions addressed theoretical knowledge on infection prevention and hand hygiene, while the third focused on supervised demonstration and re-demonstration of assigned hand hygiene techniques until competency was achieved. Practical sessions were conducted during morning shifts. Hand imprints were collected immediately before and after practice for microbial analysis.

During the study period, participants were instructed to perform the assigned hand hygiene modality during the supervised study procedures and microbial sampling sessions. Compliance with the assigned method was monitored by the research team during these observation periods to ensure correct application of the intervention.

However, outside the supervised sampling sessions, nurses continued to follow the standard infection prevention and control policies of the hospital, which may include the use of other hand hygiene methods as required for patient safety. This approach ensured that routine clinical care was not compromised while allowing controlled comparison of the assigned hand hygiene modalities during the study measurements.

### Evaluation phase

The primary outcome of interest was the reduction in microbial colony-forming units (CFU) on nurses’ hands following the assigned hand hygiene modality. Educational sessions were not intended as an experimental variable but were implemented to standardize hand hygiene practices across participants prior to microbiological comparison.

Nurses were evaluated at baseline (pre-intervention), post–1st week, post–2nd week, and post–3rd week post-intervention. Knowledge, hand hygiene performance, and microbial colonization were reassessed using the same tools. Each nurse provided six hand imprints, yielding a total of 270 samples. Microbial contamination of nurses’ hands was assessed using the hand imprint technique, in which the dominant hand was pressed onto blood agar plates for approximately 5 s. Blood agar was selected as a general-purpose culture medium to allow growth of a wide range of aerobic bacteria and to provide an overall estimate of colony-forming units (CFU) present on the hand surface.

Data were analyzed using appropriate statistical methods to compare the effectiveness of the three hand hygiene modalities in reducing microbial growth.

### Blinding and bias mitigation

No blinding was implemented in this study. Participants, observers, and microbiologists were aware of group assignments, as hand hygiene practices were directly observed and microbial samples were collected by the research team. To minimize potential bias, sample collection and microbiological analysis were standardized using consistent procedures, timing, and laboratory techniques across all groups. Nevertheless, we acknowledge that the absence of blinding may have introduced performance and observer bias, which is addressed in the Limitations section.

### Statistical analysis

Data were analyzed using IBM SPSS version 20.0 (IBM Corp., Armonk, NY). Descriptive statistics summarized categorical variables as frequencies and percentages, and quantitative variables as range, mean, standard deviation, and median. Normality was assessed using the Shapiro–Wilk test. Statistical significance was set at *p* < 0.05. Group comparisons for categorical variables were performed using the chi-square test, with Fisher’s exact or Monte Carlo correction applied when expected cell counts were low. For normally distributed quantitative variables, Student’s *t*-test and one-way ANOVA with *post-hoc* (Tukey/LSD) tests were used for comparisons between two or more groups, respectively. Repeated-measures ANOVA with Bonferroni-adjusted *post-hoc* tests assessed changes across multiple time points. Pearson’s correlation coefficient was used to examine relationships between normally distributed quantitative variables. Between-group differences in post-intervention hand hygiene practice scores were analyzed using analysis of covariance (ANCOVA), with baseline practice scores included as a covariate to adjust for initial group differences.

## Results

Table 2 summarizes the socio-demographic characteristics of nurses across the three study groups. Regarding age, 40% of nurses in Group 1 (G1) were aged 20–<30 years. In Group 2 (G2), 40% of nurses were aged 30–<40 years and ≥40 years. In Group 3 (G3), the majority (60%) were aged 30–<40 years. Females predominated in all groups, representing 73.3% of G1, 80% of G2, and 73.3% of G3. In terms of educational qualifications, the majority of nurses held a nursing diploma (G1: 80%, G2: 60%, G3: 60%). Regarding work experience, 40% of nurses in both G1 and G2 had 5–<10 years of experience, while 40% of nurses in G2 and 60% of nurses in G3 had ≥10 years of experience. Concerning infection control training, more than half of nurses in G1 (53.3%) reported attending in-service training programs within the past 6 months, whereas the majority of nurses in G2 and G3 (53.3 and 73.3%, respectively) had not attended recent infection control training.

**Table 2:** Percentage distribution of studied nurses in the three groups in relation to their socio-demographic data (*n* = 45).

Nurses’ socio-demographic data	Group 1 (*n* = 15)		Group 2 (*n* = 15)		Group 3 (*n* = 15)		χ^2^	*p*
	No.	%	No.	%	No.	%		
Age								
20 ≤ 30 year	6	**40.0**	3	20.0	2	13.3	4.458	^MC^p = 0.356
30 ≤ 40 year	4	26.7	6	**40.0**	9	**60.0**		
≥ 40 years	5	33.3	6	**40.0**	4	26.7		
Gender								
Male	4	26.7	3	20.0	4	26.7	0.350	^MC^p = 1.000
Female	11	**73.3**	12	**80.0**	11	**73.3**		
Educational qualifications								
Diploma nursing	12	**80.0**	9	**60.0**	9	**60.0**	1.800	0.407
Bachelor of nursing	3	20.0	6	40.0	6	40.0		
Master degree in nursing	0	0.0	0	0.0	0	0.0		
Doctorate degree in nursing	0	0.0	0	0.0	0	0.0		
Years of experience								
≤ 1 year	0	0.0	0	0.0	0	0.0	2.492	^MC^p = 0.697
1 ≤ 5 years	4	26.7	3	20.0	2	13.3		
5 ≤ 10 years	6	**40.0**	6	**40.0**	4	26.7		
≥ 10 years	5	33.3	6	**40.0**	9	**60.0**		
Working unit								
Hepatobiliary surgery	2	13.3	1	6.7	2	13.3	6.228	^MC^p = 1.000
Cardio thoracic surgery	2	13.3	2	13.3	1	6.7		
Vascular surgery	1	6.7	1	6.7	2	13.3		
Urology	2	13.3	1	6.7	1	6.7		
Oncology surgery	2	13.3	2	13.3	1	6.7		
Gastrointestinal surgery	1	6.7	1	6.7	2	13.3		
ENT	2	13.3	1	6.7	2	13.3		
Eye surgery	1	6.7	2	13.3	2	13.3		
Neuro surgery	1	6.7	2	13.3	1	6.7		
Colorectal surgery	1	6.7	2	13.3	1	6.7		
Receiving or attending in service training about infection control and date								
No	7	46.7	8	**53.3**	**11**	**73.3**	2.368	0.306
**Yes**	**8**	**53.3**	7	46.7	4	26.7		
3 months	2	25.0	1	14.3	0	0.0	4.972	^MC^p = 0.722
6 months	3	**37.5**	5	**71.4**	4	**100.0**		
9 months	2	25.0	1	14.3	0	0.0		
12 months	1	12.5	0	0.0	0	0.0		
Name of training								
No	7	**46.7**	8	**53.3**	11	**73.3**	6.558	^MC^p = 0.239
Infection control	7	**46.7**	7	**46.7**	3	**20.0**		
COVID-19	1	**6.7**	0	0.0	0	0.0		
Infection control and COVID-19	0	0.0	0	0.0	1	6.7		

Group 1: Hand washing only, Group 2: Alcohol based hand rub only. Group 3: Hand washing first then ABHR χ^2^: Chi square test *Statistically significant at *p* ≤ 0.05.

Supplementary Table 1 compares nurses’ overall knowledge of infection prevention and control (IPC) and hand hygiene across the three study groups over time.

At baseline, most nurses in all groups demonstrated unsatisfactory knowledge (G1: 86.7%, G2: 93.3%, G3: 86.7%). After the first week, the majority achieved satisfactory knowledge (G1: 100%, G2: 80%, G3: 100%). This improvement was largely maintained at the second week (G1: 66.7%, G2: 73.3%, G3: 73.3%). At the third week, a decline was noted in G1 and G2, with 53.3% showing unsatisfactory knowledge, whereas G3 maintained satisfactory knowledge in 86.7% of nurses.

Within-group analysis showed significant improvements in G1 between baseline and week 1 (*p* = 0.022) and baseline and week 2 (*p* = 0.005). G3 demonstrated significant improvements between baseline and weeks 1 and 2 (*p* = 0.032 and *p* < 0.001, respectively), and between weeks 1 and 2 (*p* = 0.006). A significant baseline difference was observed between G1 and G3 (*p* = 0.017).

Overall, significant improvements in knowledge were observed over time in G1 and G2 across all post-intervention periods (*p* < 0.001), while in G3 improvements were significant up to the second week. No significant differences were found between groups during post-intervention assessments.

Supplementary Table 2 presents the comparison of nurses’ hand hygiene practices among the three study groups across the study periods. At baseline, statistically significant differences were observed between Group 1 (G1) and Group 2 (G2) (*p* = 0.012), and between G1 and Group 3 (G3) (*p* < 0.001), indicating baseline imbalance between groups.

To account for these differences, analysis of covariance (ANCOVA) was performed using baseline scores as a covariate. After adjustment, between-group comparisons at post-intervention time points remained statistically significant, particularly between G1 and both G2 and G3 at the second and third weeks (*p* < 0.05). These findings suggest that differences in hand hygiene practices persisted after controlling for baseline variability; however, results should be interpreted with caution due to the initial group imbalance.

Supplementary Table 3 compares microbial colonization on nurses’ hands across the three study groups over time. No statistically significant differences were observed between groups at baseline (*p* = 0.116). However, a statistically significant difference was detected after the first week of hand hygiene practice (*p* = 0.017). Significant differences in microbial colonization were also observed among the groups at the second and third weeks post-intervention (*p* = 0.003 and *p* = 0.044, respectively).

Coagulase-negative staphylococci and *Staphylococcus aureus* were the most frequently isolated microorganisms across all assessment periods, whereas fungi and gram-negative bacteria were the least commonly identified.

Supplementary Table 4 presents the comparison of microbial colonization on nurses’ hands among the three study groups following hand hygiene procedures across the study periods. After the first and second weeks of intervention, the highest proportion of nurses with no detectable microbial growth was observed in Group 3 (hand washing followed by alcohol-based hand rub), with 100 and 86.7%, respectively. This contrasted with lower proportions in Group 2 (alcohol-based hand rub only: 60 and 6.7%) and Group 1 (hand washing only: 6.7 and 13.3%). Statistically significant differences in microbial colonization were identified among the three groups at all assessment points, including baseline and post-intervention periods (*p* = 0.005 at baseline; *p* < 0.001 at post-first and post-second weeks; *p* = 0.003 at the post-third week).

Regarding microbial identification, coagulase-negative staphylococci and *Staphylococcus aureus* were the most frequently isolated microorganisms across all study periods, while fungi and gram-negative bacteria were least commonly detected.

Table 3 presents the correlation between nurses’ knowledge and hand hygiene practices across the three study groups. Overall, correlations between knowledge and practice were weak and statistically non-significant across all groups and time points. In Group 1 (G1), a weak positive correlation was observed at baseline (*r* = 0.242, *p* = 0.384), with no significant correlations at subsequent assessments. In Group 2 (G2), a weak negative correlation was observed at baseline (*r* = −0.167, *p* = 0.551), and no significant correlations were identified post-intervention. Similarly, in Group 3 (G3), correlation coefficients were minimal and non-significant at all-time points.

**Table 3:** Correlation between Nurses Knowledge and nurses hand hygiene practice in the three studied groups (*n* = 45).

Nurses hand hygiene practice in the three studied groups			Nurses knowledge		
			Group 1 (*n* = 15)	Group 2 (*n* = 15)	Group 3 (*n* = 15)
Hand hygiene practices	Pre	*r*	0.242	0.167	0.373
		*p*	0.384	0.551	0.170
	Post 1st	*r*	0.217	–	−0.204
		*p*	0.437	–	0.467
	Post 2nd	*r*	−0.125	−0.322	0.082
		*p*	0.657	0.242	0.773
	Post 3rd	*r*	0.284	0.046	−0.006
		*p*	0.305	0.870	0.983

Group 1: Hand washing only, Group 2: Alcohol based hand rub only. Group 3: Hand washing first then alcohol-based hand rub. r: Pearson coefficient. *Statistically significant at *p* ≤ 0.05.

Given the small sample size per group, these findings should be interpreted with caution, as the analysis may be underpowered to detect meaningful associations between knowledge and practice.

## Discussion

This study examined the effectiveness of different hand hygiene modalities—hand washing alone, alcohol-based hand rub (ABHR) alone, and hand washing followed by ABHR—in reducing microbial colonization on nurses’ hands in surgical departments. The findings provide preliminary evidence that the combined hand hygiene approach may reduce microbial load more effectively under controlled conditions. However, these results must be interpreted with caution due to the small sample size, baseline differences, and quasi-experimental design.

### Interpretation of findings in relation to research hypotheses

Regarding H1, which hypothesized that nurses practicing ABHR would demonstrate lower microbial growth than those practicing hand washing alone, the results partially supported this hypothesis. Nurses in the ABHR-only group showed greater microbial reduction compared with the hand washing group during post-intervention assessments. This is consistent with previous studies showing that ABHR provides rapid, broad-spectrum antimicrobial activity, particularly against transient flora, and is more effective than hand washing when hands are not visibly soiled (13, 27). The shorter application time and convenience of ABHR may enhance adherence under ideal conditions.

In contrast, H2, which proposed that hand washing would be more effective than ABHR, was not supported. Hand washing reduced microbial counts relative to baseline but was less effective than ABHR and markedly less effective than the combined method. This may be attributable to variations in technique, insufficient duration, or inadequate mechanical friction during hand washing (12, 28).

The strongest support was observed for H3, hypothesizing that hand washing followed by ABHR would result in the lowest microbial colonization. Group 3 consistently demonstrated the highest proportion of nurses with no detectable microbial growth. While this finding suggests a potential additive benefit of sequential application, it does not override WHO guidance, which recommends ABHR as the standard method and hand washing only when hands are visibly soiled or for spore-forming pathogens. Moreover, the combined method is time- and resource-intensive, which may reduce compliance in busy clinical settings (29, 30). These factors must be considered when interpreting the practical relevance of the combined approach.

### Microbiological profile of hand contamination

Coagulase-negative staphylococci and *Staphylococcus aureus* predominated across all assessment periods, consistent with the normal skin flora of healthcare workers and their frequent association with healthcare-associated infections (31). The relatively low detection of fungi and gram-negative bacteria aligns with prior research indicating their reduced survival on intact skin and susceptibility to routine hand hygiene measures (23). It should be noted that microbial sampling was limited to the palmar surface using blood agar; therefore, results may not fully reflect microbial loads in interdigital spaces, nail beds, or dorsum of the hand.

### Knowledge, practice, and sustainability of hand hygiene behavior

Although nurses’ knowledge and hand hygiene practices improved after the intervention, declines were observed during later follow-up periods, especially in the hand washing and ABHR-only groups. This suggests that education alone may yield short-term improvements but is insufficient for sustained behavior change (32, 16).

The study did not detect statistically significant correlations between nurses’ knowledge and hand hygiene practice. Given the small sample size (*n* = 15 per group), these findings are inconclusive and likely reflect insufficient statistical power rather than a true lack of association. Therefore, this result should not be interpreted as evidence that knowledge alone does not guarantee adherence. Other factors, including workload, staffing levels, availability of supplies, institutional culture, and leadership support, likely influence hand hygiene behavior (33–35).

### Implications for nursing practice

The findings of this study have practical implications for infection prevention and control in clinical settings, but should be interpreted cautiously due to the small sample size, short study duration, and baseline differences between groups.

First, while sequential hand washing followed by alcohol-based hand rub (ABHR) demonstrated the greatest reduction in microbial colonization under controlled conditions, WHO guidelines remain the standard recommendation: ABHR for routine hand hygiene and hand washing when hands are visibly soiled or for spore-forming pathogens. The combined approach may offer additional microbial reduction, but its time- and resource-intensive nature may limit routine applicability in busy clinical environments.

Second, hand hygiene education should extend beyond single-session training models. Continuous education programs, periodic competency assessments, and ongoing feedback are essential to sustain improvements in knowledge and practice. Visual reminders, audit-and-feedback systems, and role modeling by senior staff can further enhance compliance.

Third, healthcare organizations must ensure the consistent availability of hand hygiene resources, including sinks, soap, disposable towels, and ABHR at point-of-care locations. System-level support is critical to enable nurses to perform hand hygiene correctly and consistently, particularly under high workload conditions.

Finally, integrating regular microbiological surveillance and observation-based audits into infection control programs can reinforce accountability and provide objective feedback on hand hygiene effectiveness, thereby enhancing nurses’ awareness of the direct impact of their practices on microbial transmission and patient safety.

## Conclusion

Hand hygiene reduces microbial colonization on nurses’ hands, with effectiveness varying by method. In this study, hand washing followed by alcohol-based hand rub showed the greatest reduction under controlled conditions. Short-term improvements in knowledge and practice were observed but were not consistently sustained, highlighting the need for ongoing training and institutional support. WHO-recommended practices remain the standard, and feasibility considerations must guide implementation in clinical settings.

### Limitations of the study and future directions

This study has several important limitations. First, the non-random allocation of participants using an alternation method introduces potential selection bias, meaning the study cannot be considered a true randomized controlled trial. Second, baseline imbalances in hand hygiene practice between groups may have influenced post-intervention comparisons, even though analyses were adjusted using ANCOVA. Third, the inclusion of a pre-intervention educational program for all groups confounds the ability to attribute changes solely to the hand hygiene modality.

Additional limitations include the single-center design and small sample size, which limit generalizability, and the short follow-up period, restricting assessment of long-term sustainability of hand hygiene practices. Direct observation may have introduced a Hawthorne effect, and variations in workload and unit characteristics were not controlled. Microbiological analysis was limited to palmar colony counts on blood agar, excluding interdigital spaces, nail beds, dorsum of the hand, and selective media for gram-negative bacteria or fungi, and did not include molecular or resistance profiling. Finally, blinding of participants and outcome assessors was not implemented, increasing the risk of observer and performance bias.

Future studies should employ true randomization with concealed allocation, larger multicenter samples, and longer follow-up periods to enhance generalizability and assess sustainability. Integrating continuous education, audit-and-feedback systems, and organizational support factors is recommended to better understand and optimize hand hygiene compliance. Advanced microbiological techniques, including comprehensive sampling and resistance profiling, could provide deeper insights into pathogen transmission and control.

## Data Availability

The raw data supporting the conclusions of this article will be made available by the authors, without undue reservation.
