# Supplementary material for: Comparative effectiveness of hand hygiene modalities in reducing microbial colonization on nurses’ hands in surgical settings in Jordan
Source: Front Public Health. 2026 May 8;14:1794667. doi: 10.3389/fpubh.2026.1794667 (PMC13194477; doi:10.3389/fpubh.2026.1794667)
Supplement: Supplementary file 1 [file Supplementary_file_1.docx]

**Supplementary Table 1: Comparison between overall nurses’ knowledge regarding to (IPC and hand hygiene) in the three studied groups along the periods of study (n=45)**

| **Knowledge of IPC and hand hygiene** | **Group 1(n = 15)** | | | | | | | | **Group 2(n = 15)** | | | | | | | | **Group 3(n = 15)** | | | | | | | | **Significant between groups** | | | |
| --- | --- | --- | --- | --- | --- | --- | --- | --- | --- | --- | --- | --- | --- | --- | --- | --- | --- | --- | --- | --- | --- | --- | --- | --- | --- | --- | --- | --- |
|  | **Pre** | | **Post 1^st^** | | **Post 2^nd^** | | **Post 3^rd^** | | **Pre** | | **Post 1^st^** | | **Post 2^nd^** | | **Post 3^rd^** | | **Pre** | | **Post 1^st^** | | **Post 2^nd^** | | **Post 3^rd^** | |  |  |  |  |
|  | **No.** | **%** | **No.** | **%** | **No.** | **%** | **No.** | **%** | **No.** | **%** | **No.** | **%** | **No.** | **%** | **No.** | **%** | **No.** | **%** | **No.** | **%** | **No.** | **%** | **No.** | **%** | **Pre** | **Post 1^st^** | **Post 2^nd^** | **Post 3^rd^** |
| < 90 % Unsatisfactory | 13 | **86.7** | 0 | 0.0 | 5 | 33.3 | 8 | **53.3** | 14 | **93.3** | 3 | 20.0 | 4 | 26.7 | 8 | **53.3** | 13 | **86.7** | 0 | 0.0 | 4 | 26.7 | 13 | **86.7** | χ^2^=0.636 (^MC^p= 1.000) | χ^2^=4.441 (^MC^p= 0.095) | χ^2^=0.309 (^MC^p= 1.000) | χ^2^=4.849 (0.089) |
| ≥90% satisfactory | 2 | 13.3 | 15 | **100.0** | 10 | **66.7** | 7 | 46.7 | 1 | 6.7 | 12 | **80.0** | 11 | **73.3** | 7 | 46.7 | 2 | 13.3 | 15 | **100.0** | 11 | **73.3** | 2 | 13.3 |  |  |  |  |
| **Total score** |  | |  | |  | |  | |  | |  | |  | |  | |  | |  | |  | |  | |  |  |  |  |
| Min. – Max. | 16.0 – 27.0 | | 27.0 – 41.0 | | 24.0 – 28.0 | | 21.0 – 28.0 | | 18.0 – 27.0 | | 25.0 – 30.0 | | 22.0 – 29.0 | | 20.0 – 28.0 | | 19.0 – 27.0 | | 27.0 – 30.0 | | 23.0 – 29.0 | | 21.0 – 27.0 | | F=4.722^*^ (0.014^*^) | F=2.782 (0.073) | F=0.382 (0.685) | F=1.154 (0.325) |
| Mean ± SD | 20.27 ± 3.37 | | 29.40 ± 3.33 | | 26.40 ± 1.12 | | 25.53 ± 2.23 | | 20.80 ± 2.43 | | 27.53 ± 1.41 | | 26.27 ± 2.25 | | 25.20 ± 2.48 | | 23.20 ± 2.46 | | 28.27 ± 1.10 | | 26.80 ± 1.66 | | 24.33 ± 1.95 | |  |  |  |  |
| **Percent score** |  | |  | |  | |  | |  | |  | |  | |  | |  | |  | |  | |  | |  |  |  |  |
| Min. – Max. | 53.33–90.0 | | 90.0 – 100 | | 80.0 – 93.33 | | 70.0 – 93.33 | | 60.0 – 90.0 | | 83.33–100.0 | | 73.33–96.67 | | 66.67–93.33 | | 63.33 – 90.0 | | 90.0 – 100.0 | | 76.67–96.67 | | 70.0 – 90.0 | |  |  |  |  |
| Mean ± SD | 67.56±11.23 | | 98.0 ± 11.11 | | 88.0 ± 3.74 | | 85.11 ± 7.44 | | 69.33 ± 8.09 | | 91.78 ± 4.69 | | 87.56 ± 7.50 | | 84.0 ± 8.28 | | 77.33 ± 8.18 | | 94.22 ± 3.67 | | 89.33 ± 5.52 | | 81.11 ± 6.51 | |  |  |  |  |
| **Sig. with pre period** |  | | **<0.001^*^** | | **<0.001^*^** | | **0.001^*^** | |  | | **<0.001^*^** | | **<0.001^*^** | | **0.001^*^** | |  | | **<0.001^*^** | | **0.004^*^** | | **1.000** | | p_1_=0.860  **p_2_=0.017**^*^  p_3_=0.058 | – | – | – |
| **Sig. with post1,2,3** | **p_4_=0.022^*^, p_5_=0.005^*^,** p_6_=1.000 | | | | | | | | p_4_=0.603, p_5_=0.058, p_6_=1.000 | | | | | | | | **p_4_=0.032^*^, p_5_<0.001^*^,p_6_=0.006^*^** | | | | | | | |  |  |  |  |

**Group 1: Hand washing only, Group 2: Alcohol based hand rub only, Group 3: Hand washing first then alcohol-based hand rub**

**Pre=Preprogram, Post1=Post 1^st^ week program, Post2=Post 2^nd^ week program, Post3=Post 3^rd^ week program**

χ^2^: **Chi square test** MC: **Monte Carlo**

**F**: **F for One way ANOVA test**, pairwise comparison bet. each 2 groups were done using **Post Hoc Test (Tukey)**

p_0_: p value for Post Hoc test (**adjusted Bonferroni**) for comparing between **Pre** and **each other periods**

p: p value for comparing between the **three studied groups**  p_1_: p value for comparing between **group 1** and **group 2**

p_2_: p value for comparing between **group 1** and **group 3** p_3_: p value for comparing between **group 2** and **group 3**

p_4_: p value for Post Hoc test (**adjusted Bonferroni**) for comparing between **Pre** and **Post1**

p_5_: p value for Post Hoc test (**adjusted Bonferroni**) for comparing between **Pre** and **Post2**

p_6_: p value for Post Hoc test (**adjusted Bonferroni**) for comparing between **Post1** and **Post2**
*: Statistically significant at p ≤ 0.05

**Supplementary Table 2: Comparison between nurses’ hand hygiene practices in the three studied groups along the periods of study (n=45)**

| **hand hygiene practice  % score** | **Group 1(n = 15)** | | | | | | | | **Group 2(n = 15)** | | | | | | | | **Group 3(n = 15)** | | | | | | | | **Significant between groups** | | | |
| --- | --- | --- | --- | --- | --- | --- | --- | --- | --- | --- | --- | --- | --- | --- | --- | --- | --- | --- | --- | --- | --- | --- | --- | --- | --- | --- | --- | --- |
|  | **Pre** | | **Post 1^st^** | | **Post 2^nd^** | | **Post 3^rd^** | | **Pre** | | **Post 1^st^** | | **Post 2^nd^** | | **Post 3^rd^** | | **Pre** | | **Post 1^st^** | | **Post 2^nd^** | | **Post 3^rd^** | |  |  |  |  |
|  | **No.** | **%** | **No.** | **%** | **No.** | **%** | **No.** | **%** | **No.** | **%** | **No.** | **%** | **No.** | **%** | **No.** | **%** | **No.** | **%** | **No.** | **%** | **No.** | **%** | **No.** | **%** | **Pre** | **Post 1^st^** | **Post 2^nd^** | **Post 3^rd^** |
| <90% unsatisfactory | 15 | **100.0** | 0 | 0.0 | 8 | **53.3** | 14 | **93.3** | 15 | **100.0** | 0 | 0.0 | 3 | 20.0 | 13 | **86.7** | 15 | **100.0** | 1 | 6.7 | 2 | 13.3 | 11 | **73.3** | − | χ^2^=1.862 (^MC^p=1.000 | χ^2^=6.112 (^MC^p=0.066 | χ^2^=2.166 (^MC^p=0.460 |
| ≥90% satisfactory | 0 | 0.0 | 15 | **100.0** | 7 | 46.7 | 1 | 6.7 | 0 | 0.0 | 15 | **100.0** | 12 | **80.0** | 2 | 13.3 | 0 | 0.0 | 14 | **93.3** | 13 | **86.7** | 4 | 26.7 |  |  |  |  |
| Min. – Max. | 25.0– 50.0 | | 91.67–100.0 | | 66.67–100.0 | | 58.33–91.67 | | 44.44–66.67 | | 100–100 | | 88.89–100.0 | | 55.56–100.0 | | 47.62–71.43 | | 71.43–100.0 | | 85.71–100.0 | | 71.43–100.0 | | F=9.173^*^ (p<0.001^*^) | F=1.126  (p=0.334) | F=16.090^*^ (p<0.001^*^) | F=5.208^*^ (p=0.010^*^) |
| Mean ± SD | 45.0± 7.59 | | 98.33±3.45 | | 83.89±10.19 | | 72.22±8.13 | | 53.33 ± 8.61 | | 100±0.0 | | 97.78 ± 4.60 | | 82.22±12.46 | | 56.51 ± 6.46 | | 97.46±7.39 | | 95.56±5.54 | | 83.17±9.84 | |  |  |  |  |
| **Sig. with pre period (p_0_)** |  | | **<0.001^*^** | | **<0.001^*^** | | **<0.001^*^** | |  | | **<0.001^*^** | | **<0.001^*^** | | **<0.001^*^** | |  | | **<0.001^*^** | | **<0.001^*^** | | **<0.001^*^** | | **p_1_=0.012^*^,**  **p_2_<0.001^*^,**  p_3_=0.493 | – | **p_1_<0.001^*^,**  **p_2_<0.001^*^,**  p_3_=0.678 | **p_1_=0.029^*^,**  **p_2_=0.015^*^,**  p_3_=0.965 |
| **Sig. with post1,2,3** |  | | **p_4_<0.001^*^, p_5_<0.001^*^, p_6_=0.021^*^** | | | | | |  | | **p_4_=0.495, p_5_<0.001^*^, p_6_=0.002^*^** | | | | | |  | | **p_4_=1.000, p_5_=0.008^*^, p_6_=0.001^*^** | | | | | |  |  |  |  |
|  |  | |  |  |  |  |  |  |  | |  |  |  |  |  |  |  | |  |  |  |  |  |  |  |  |  |  |

**Group 1: Hand washing only, Group 2: Alcohol based hand rub only, Group 3: Hand washing first then alcohol-based hand rub**

**Pre=Preprogram, Post1=Post 1^st^ week program, Post2=Post 2^nd^ week program, Post3=Post 3^rd^ week program**

χ^2^: **Chi square test** MC: **Monte Carlo**

**F**: **F for One way ANOVA test**, pairwise comparison bet. each 2 groups were done using **Post Hoc Test (Tukey)**

p: p value for comparing between the **three studied groups**  p_1_: p value for comparing between **group 1** and **group 2**

p_2_: p value for comparing between **group 1** and **group 3** p_3_: p value for comparing between **group 2** and **group 3**

p_0_: p value for Post Hoc test (**adjusted Bonferroni**) for comparing between **Pre** and **each other periods**

p_4_: p value for Post Hoc test (**adjusted Bonferroni**) for comparing between **Post1** and **Post2**

p_5_: p value for Post Hoc test (**adjusted Bonferroni**) for comparing between **Post1** and **Post3**

p_6_: p value for Post Hoc test (**adjusted Bonferroni**) for comparing between **Post2** and **Post3**
*: Statistically significant at p ≤ 0.05

**Supplementary Table 3: Comparison between three studied groups regarding to microorganisms' colonization on nurses' hands pre hand hygiene along the periods of hand hygiene practice (n=45)**

| **pre-Hand hygiene** | **Group 1** | | | | | | | | **Group 2** | | | | | | | | **Group 3** | | | | | | | | **χ^2^ (^MC^p)** | | | |
| --- | --- | --- | --- | --- | --- | --- | --- | --- | --- | --- | --- | --- | --- | --- | --- | --- | --- | --- | --- | --- | --- | --- | --- | --- | --- | --- | --- | --- |
|  | **Pre** | | **Post 1^st^** | | **Post 2^nd^** | | **Post 3^rd^** | | **Pre** | | **Post 1^st^** | | **Post 2^nd^** | | **Post 3^rd^** | | **Pre** | | **Post 1^st^** | | **Post 2^nd^** | | **Post 3^rd^** | |  |  |  |  |
|  | **No.** | **%** | **No.** | **%** | **No.** | **%** | **No.** | **%** | **No.** | **%** | **No.** | **%** | **No.** | **%** | **No.** | **%** | **No.** | **%** | **No.** | **%** | **No.** | **%** | **No.** | **%** | **Pre** | **Post 1^st^** | **Post 2^nd^** | **Post 3^rd^** |
| **Number of colonized microorganisms** |  |  |  |  |  |  |  |  |  |  |  |  |  |  |  |  |  |  |  |  |  |  |  |  |  |  |  |  |
| - Zero CFU (no microbial growth) | 0 | 0.0 | 0 | 0.0 | 0 | 0.0 | 0 | 0.0 | 0 | 0.0 | 0 | 0.0 | 0 | 0.0 | 0 | 0.0 | 0 | 0.0 | 0 | 0.0 | 0 | 0.0 | 0 | 0.0 | 4.591 (0.116) | 5.975 (0.071) | **11.534^*^**  **(0.003^*^)** | **7.885^*^  (0.044^*^)** |
| - < 50 CFU (mild microbial growth) | 0 | 0.0 | 0 | 0.0 | 0 | 0.0 | 1 | 6.7 | 0 | 0.0 | 0 | 0.0 | 0 | 0.0 | 0 | 0.0 | 0 | 0.0 | 0 | 0.0 | 0 | 0.0 | 0 | 0.0 |  |  |  |  |
| - 50 to < 100 CFU (moderate microbial growth) | 6 | 40.0 | 3 | 20.0 | 10 | 66.7 | 7 | 46.7 | 3 | 20.0 | 5 | 33.3 | 6 | 40.0 | 2 | 13.3 | 1 | 6.7 | 0 | 0.0 | 1 | 6.7 | 2 | 13.3 |  |  |  |  |
| - ≥ 100 CFU (heavy microbial growth) | 9 | 60.0 | 12 | 80.0 | 5 | 33.3 | 7 | 46.7 | 12 | 80.0 | 10 | 66.7 | 9 | 60.0 | 13 | 86.7 | 14 | 93.3 | 15 | 100.0 | 14 | 93.3 | 13 | 86.7 |  |  |  |  |
| - Negative | 0 | 0.0 | 0 | 0.0 | 0 | 0.0 | 0 | 0.0 | 0 | 0.0 | 0 | 0.0 | 0 | 0.0 | 0 | 0.0 | 0 | 0.0 | 0 | 0.0 | 0 | 0.0 | 0 | 0.0 |  |  |  |  |
| **Microorganisms’ identification** |  |  |  |  |  |  |  |  |  |  |  |  |  |  |  |  |  |  |  |  |  |  |  |  |  |  |  |  |
| - Coagulase –negative staphylococci (CoNS) | 15 | 100.0 | 15 | 100.0 | 15 | 100.0 | 15 | 100.0 | 15 | 100.0 | 15 | 100.0 | 15 | 100.0 | 15 | 100.0 | 15 | 100.0 | 15 | 100.0 | 15 | 100.0 | 15 | 100.0 | **–** | – | **–** | **–** |
| - Staphylococci aureus | 15 | 100.0 | 15 | 100.0 | 15 | 100.0 | 15 | 100.0 | 15 | 100.0 | 14 | 93.3 | 15 | 100.0 | 15 | 100.0 | 13 | 86.7 | 15 | 100.0 | 15 | 100.0 | 15 | 100.0 | 2.812 (0.324) | 1.862 (1.000) | **–** | **–** |
| - Fungi | 2 | 13.3 | 0 | 0.0 | 2 | 13.3 | 1 | 6.7 | 3 | 20.0 | 1 | 6.7 | 0 | 0.0 | 0 | 0.0 | 3 | 20.0 | 1 | 6.7 | 2 | 13.3 | 0 | 0.0 | 0.441 (1.000) | 1.288 (1.000) | 2.241  (0.525) | 1.862  (1.000) |
| - Gram-negative bacteria (GNB) | 1 | 6.7 | 2 | 13.3 | 2 | 13.3 | 0 | 0.0 | 2 | 13.3 | 3 | 20.0 | 0 | 0.0 | 2 | 13.3 | 3 | 20.0 | 1 | 6.7 | 2 | 13.3 | 1 | 6.7 | 1.184  (0.859) | 1.184 (0.859) | 2.241  (0.525) | 1.957 (0.768) |

**Group 1: Hand washing only, Group 2: Alcohol based hand rub only, Group 3: Hand washing first then alcohol-based hand rub**

**Pre=Preprogram, Post1=Post 1^st^ week program, Post2=Post 2^nd^ week program, Post3=Post 3^rd^ week program**

χ^2^: **Chi square test** MC: **Monte Carlo**

p: p value for comparing between the **three studied groups**
*: Statistically significant at p ≤ 0.05

**Supplementary Table 4: Comparison between three studied groups regarding to microorganisms' colonization on nurses' hands post hand hygiene along the periods of study (n=45)**

| **After Hand wash** | **Group 1** | | | | | | | | | **Group 2** | | | | | | | | | **Group 3** | | | | | | | | | | | | | **χ^2^ (^MC^p)** | | | | |
| --- | --- | --- | --- | --- | --- | --- | --- | --- | --- | --- | --- | --- | --- | --- | --- | --- | --- | --- | --- | --- | --- | --- | --- | --- | --- | --- | --- | --- | --- | --- | --- | --- | --- | --- | --- | --- |
|  | **Pre** | | **Post 1^st^** | | **Post 2^nd^** | | **Post 3^rd^** | | | **Pre** | | **Post 1^st^** | | **Post 2^nd^** | | **Post 3^rd^** | | **Pre** | | | | **Post 1^st^** | | | **Post 2^nd^** | | | **Post 3^rd^** | | |  | | | | |  |
|  | **No.** | **%** | **No.** | **%** | **No.** | **%** | **No.** | **%** | **No.** | | **%** | **No.** | **%** | **No.** | **%** | **No.** | **%** | **No.** | | **%** | **No.** | | **%** | **No.** | | **%** | **No.** | | **%** | **Pre** | | | **Post 1^st^** | **Post 2^nd^** | **Post 3^rd^** |  |
| **NO of colonized microorganisms** |  |  |  |  |  |  |  |  |  | |  |  |  |  |  |  |  |  | |  |  | |  |  | |  |  | |  |  | | |  |  |  |  |
| - Zero CFU (no microbial growth) | 0 | 0.0 | 1 | 6.7 | 2 | 13.3 | 1 | 6.7 | 0 | | 0.0 | 9 | **60.0** | 1 | 6.7 | 0 | 0.0 | 0 | | 0.0 | 15 | | **100.0** | 13 | | **86.7** | 6 | | 40.0 | 13.395^*^ **(0.005^*^)** | | | 30.357^*^  **(<0.001^*^)** | 26.607^*^ **(<0.001^*^)** | 12.577^*^ **(0.003^*^)** |  |
| - < 50 CFU (mild microbial growth) | 1 | 6.7 | 13 | **86.7** | 12 | **80.0** | 10 | **66.7** | 0 | | 0.0 | 6 | 40.0 | 14 | **93.3** | 14 | **93.3** | 3 | | 20.0 | 0 | | 0.0 | 2 | | 13.3 | 9 | | **60.0** |  |  |  |  |  |  |  |
| - 50 to < 100 CFU (moderate microbial growth) | 8 | 53.3 | 1 | 6.7 | 1 | 6.7 | 4 | 26.7 | 7 | | 46.7 | 0 | 0.0 | 1 | 6.7 | 1 | 6.7 | 12 | | **80.0** | 0 | | 0.0 | 0 | | 0.0 | 0 | | 0.0 |  |  |  |  |  |  |  |
| - ≥ 100 CFU (heavy microbial growth) | 6 | **40.0** | 0 | 0.0 | 0 | 0.0 | 0 | 0.0 | 8 | | **53.3** | 0 | 0.0 | 0 | 0.0 | 0 | 0.0 | 0 | | 0.0 | 0 | | 0.0 | 0 | | 0.0 | 0 | | 0.0 |  |  |  |  |  |  |  |
| - Negative | 0 | 0.0 | 0 | 0.0 | 0 | 0.0 | 0 | 0.0 | 0 | | 0.0 | 0 | 0.0 | 0 | 0.0 | 0 | 0.0 | 0 | | 0.0 | 0 | | 0.0 | 0 | | 0.0 | 0 | | 0.0 |  |  |  |  |  |  |  |
| **Microorganisms’ identification** |  |  |  |  |  |  |  |  |  | |  |  |  |  |  |  |  |  | |  |  | |  |  | |  |  | |  |  | | |  |  |  |  |
| - Coagulase –negative staphylococci (CoNS) | 15 | 100.0 | 14 | 93.3 | 13 | 86.7 | 14 | 93.3 | 15 | | 100.0 | 6 | 40.0 | 14 | 93.3 | 15 | 100.0 | 15 | | 100.0 | 0 | | 0.0 | 2 | | 13.3 | 9 | | 60.0 | **–** | | | 26.640^*^ (<0.001^*^) | 25.797^*^  (<0.001^*^) | 8.875^*^ (0.010^*^) |  |
| - Staphylococci aureus | 15 | 100.0 | 14 | 93.3 | 12 | 80.0 | 14 | 93.3 | 15 | | 100.0 | 6 | 40.0 | 14 | 93.3 | 15 | 100.0 | 14 | | 93.3 | 0 | | 0.0 | 1 | | 6.7 | 8 | | 53.3 | 1.862  (1.000) | | | 26.640^*^ (<0.001^*^) | 27.222^*^ (<0.001^*^) | 11.275^*^ (0.002^*^) |  |
| - Fungi | 2 | 13.3 | 0 | 0.0 | 1 | 6.7 | 1 | 6.7 | 3 | | 20.0 | 0 | 0.0 | 0 | 0.0 | 0 | 0.0 | 1 | | 6.7 | 0 | | 0.0 | 0 | | 0.0 | 0 | | 0.0 | 1.184 (0.862) | | | – | 1.862 (1.000) | 1.862 (1.000) |  |
| - Gram-negative bacteria (GNB) | 1 | 6.7 | 1 | 6.7 | 2 | 13.3 | 1 | 6.7 | 1 | | 6.7 | 0 | 0.0 | 0 | 0.0 | 1 | 6.7 | 3 | | 20.0 | 0 | | 0.0 | 1 | | 6.7 | 1 | | 6.7 | 1.596  (0.595) | | | 1.862 (1.000) | 1.957 (0.769) | 0.433 (1.000) |  |
| - Negative | 0 | 0.0 | 1 | 6.7 | 2 | 13.3 | 1 | 6.7 | 0 | | 0.0 | 9 | 60.0 | 1 | 6.7 | 0 | 0.0 | 0 | | 0.0 | 15 | | 100.0 | 13 | | 86.7 | 6 | | 40.0 | – | | | 26.640^*^ (<0.001^*^) | 25.312^*^ (<0.001^*^) | 8.875^*^ (0.010^*^) |  |

**Group 1: Hand washing only, Group 2: Alcohol based hand rub only, Group 3: Hand washing first then alcohol-based hand rub**

**Pre=Preprogram, Post1=Post 1^st^ week program, Post2=Post 2^nd^ week program, Post3=Post 3^rd^ week program**

χ^2^: **Chi square test** MC: **Monte Carlo** p: p value for comparing between the **three studied groups**  *: Statistically significant at p ≤ 0.05
